# Differential Response of Grapevine to Infection with ‘*Candidatus* Phytoplasma solani’ in Early and Late Growing Season through Complex Regulation of mRNA and Small RNA Transcriptomes

**DOI:** 10.3390/ijms22073531

**Published:** 2021-03-29

**Authors:** Marina Dermastia, Blaž Škrlj, Rebeka Strah, Barbara Anžič, Špela Tomaž, Maja Križnik, Christina Schönhuber, Monika Riedle-Bauer, Živa Ramšak, Marko Petek, Aleš Kladnik, Nada Lavrač, Kristina Gruden, Thomas Roitsch, Günter Brader, Maruša Pompe-Novak

**Affiliations:** 1 National Institute of Biology, 1000 Ljubljana, Slovenia; rebeka.strah@gmail.com (R.S.); barbara.anzic@gmail.com (B.A.); spela.tomaz@nib.si (Š.T.); maja.kriznik@nib.si (M.K.); ziva.ramsak@nib.si (Ž.R.); marko.petek@nib.si (M.P.); kristina.gruden@nib.si (K.G.); marusa.pompe.novak@nib.si (M.P.-N.); 2 Jožef Stefan Institute, 1000 Ljubljana, Slovenia; blaz.skrlj@ijs.si (B.Š.); nada.lavrac@ijs.si (N.L.); 3 Jožef Stefan International Postgraduate School, 1000 Ljubljana, Slovenia; 4 Bioresources Unit, Austrian Institute of Technology, 3430 Tulln, Austria; christina.schoenhuber@gmail.com (C.S.); guenter.brader@ait.ac.at (G.B.); 5 Federal College and Research Institute for Viticulture and Pomology, 3400 Klosterneuburg, Austria; monika.riedle-bauer@weinobst.at; 6 Department of Biology, Biotechnical Faculty, University of Ljubljana, 1000 Ljubljana, Slovenia; ales.kladnik@bf.uni-lj.si; 7 Department of Plant and Environmental Sciences, University of Copenhagen, 2630 Taastrup, Denmark; roitsch@plen.ku.dk; 8 School of Viticulture and Enology, University of Nova Gorica, 5271 Vipava, Slovenia

**Keywords:** ‘*Candidatus* Phytoplasma solani’, grapevine, bois noir, RNA-Seq, sRNA-Seq, miRNA, phasiRNA, hormones, interaction network

## Abstract

Bois noir is the most widespread phytoplasma grapevine disease in Europe. It is associated with ‘*Candidatus* Phytoplasma solani’, but molecular interactions between the causal pathogen and its host plant are not well understood. In this work, we combined the analysis of high-throughput RNA-Seq and sRNA-Seq data with interaction network analysis for finding new cross-talks among pathways involved in infection of grapevine cv. Zweigelt with ‘*Ca*. P. solani’ in early and late growing seasons. While the early growing season was very dynamic at the transcriptional level in asymptomatic grapevines, the regulation at the level of small RNAs was more pronounced later in the season when symptoms developed in infected grapevines. Most differentially expressed small RNAs were associated with biotic stress. Our study also exposes the less-studied role of hormones in disease development and shows that hormonal balance was already perturbed before symptoms development in infected grapevines. Analysis at the level of communities of genes and mRNA-microRNA interaction networks revealed several new genes (e.g., expansins and cryptdin) that have not been associated with phytoplasma pathogenicity previously. These novel actors may present a new reference framework for research and diagnostics of phytoplasma diseases of grapevine.

## 1. Introduction

Bois noir is the most widespread phytoplasma disease of grapevines (*Vitis vinifera* L.) in Europe and can lead regionally to losses of up to 50% [1]. Its causal agent is ‘*Candidatus* Phytoplasma solani’ from the stolbur group 16SrXII-A [2]. The interactions between grapevine and ‘*Ca*. P. solani’ have been extensively studied and are reviewed elsewhere [3]. Several developmental processes and metabolic pathways in the host plant are affected by infection as revealed at the transcriptional, protein, and metabolic levels [4,5,6,7,8]. In infected grapevines, several steps in photosynthesis are repressed during infection. There is growing evidence that feedback inhibition of photosynthesis results in leaf yellowing (i.e., chlorosis) because of carbohydrate accumulation in the source leaves [9,10,11,12,13,14]. As was proposed early in phytoplasma research, this accumulation is a consequence of the manipulation of the host metabolism by the phytoplasmas, which can turn infected plant tissues into a carbohydrate sink that provides phytoplasmas with hexoses [8,15,16]. Several studies have analyzed the expression of genes involved in carbohydrate metabolism and their enzyme products and sugar metabolites upon infection of grapevines with ‘*Ca*. P. solani’ [8,16,17,18,19,20]. As a sign of stress conditions, grapevines infected with ‘*Ca*. P. solani’ accumulate several amino acids, including serine, glycine, valine, leucine, alanine, β-alanine, threonine, aspartate, pyroglutamate, and proline [17]. In addition, infection with ‘*Ca*. P. solani’ changes the flavonoid pathways in grapevine [21,22,23,24,25,26]. Although evidence points to important roles of the plant hormones in the signaling networks involved in grapevine responses to ‘*Ca*. P. solani’, the underlying molecular mechanisms of these interactions remain poorly understood [27].

While the main plant processes in which phytoplasmas are involved are now recognized, information is scarce in terms of fine-tuning the expression of the regulators involved, such as transcription factors and small RNAs (sRNAs). The combination of these factors defines the genetic regulatory circuits of transcriptional control [28,29]. sRNAs are involved in multiple cellular processes, and they modulate the expression of other regulators, including transcription factors [28,30]. In plants, two types of sRNAs regulate posttranscriptional gene expression, microRNAs (miRNAs) and phased small-interfering RNAs (phasiRNAs), although their targets are not well defined. However, several conserved and species-specific miRNAs have been identified in grapevines [31]. It has also been shown that miRNAs have an important role in host plant responses to phytoplasma infections. They have been identified in phytoplasma-infected Mexican lime [32], *Ziziphus jujuba* [33], mulberry [34], and paulownia [35], together with their putative targets, which are genes involved in plant morphology, signaling, nutrient homeostasis, environmental stress responses, and hormonal metabolism and regulation. In addition, several miRNAs that respond to phytoplasma infection have been identified in grapevines infected with Flavescence dorée phytoplasmas [36] and with ‘*Ca*. P. asteris’ [37].

New high-throughput technologies such as whole transcriptome sequencing allow simultaneous analysis of multiple gene expression snapshots of the same organism or of its tissues. These can be used for more accurate analysis of time-dependent phenomena, such as phytoplasma pathogenesis. In the present study, we addressed the temporal dynamics of the responses of grapevine cv. Zweigelt (Rotburger) to infection with ‘*Ca*. P. solani’ under natural vineyard conditions in leaf-vein-enriched samples and analyzed gene expression by RNA-Seq and sRNA profiles by sRNA-Seq.

As molecules in the cell seldom work completely independently, several interaction network-based approaches have been adopted as tools of choice to study their interconnectivity [38]. In a study parallel to the present one, we developed methods that can operate with such information-rich structures and applied these to modeling the bois noir disease [39]. The network inference we described offers an exploration of temporal network dynamics at the level of communities that can be revealed from RNA-Seq data. This method was developed on grapevine cv. Zweigelt infected with ‘*Ca*. P. solani’ late in the growing season. In the present study, we aimed to apply this method to grapevines sampled early in the growing season prior to symptom development, which has not been investigated in detail to date, and to perform a comparative analysis with the results of the late growing season. In addition, to reveal hierarchical interactions and co-regulation among different RNA classes involved in these grapevine infections, data-driven bioinformatics approaches were used to analyze the genome-wide data obtained over two consecutive growing seasons. We have identified complex regulatory networks that provide us with a reference framework for detailed studies of the regulation of the main processes in grapevines infected with ‘*Ca*. P. solani’.

## 2. Results and Discussion

### 2.1. mRNAs and sRNAs Show Differential Temporal Involvement in Grapevine Infected with ‘Ca. P. solani’

High-throughput RNA-Seq (Table S1) and sRNA-Seq (Table S2) were performed on leaf-vein-enriched samples collected in the early and late growing seasons in 2017; for mRNAs, this was repeated again early in the season of 2020. For the 2017 experiment, the grapevines were chosen based on checking for phytoplasma infection the previous year, as presumably uninfected and infected with ‘*Ca*. P. solani’. The presumed infected grapevines were asymptomatic at the time of the first sampling in the early growing season. However, they developed symptoms over the growing season, and the phytoplasma infection with a nettle type (CPsM4_At1 [40]) was confirmed at the time of the second sampling in the late growing season. The same grapevines were sampled again in the early growing season of 2020. In 2020, the grapevines did not develop symptoms over the summer, and phytoplasmas were not detected in samples from these grapevines later in the growing season, which suggested that these grapevines had already recovered from their previous infection or were in the recovery process [41]. Moreover, the RNA-Seq analysis of samples from these grapevines revealed that there was not even a single differentially expressed gene defined between the recovered and uninfected grapevines (Table S1), which indicated that the recovery appeared to have occurred previously, in 2018 or 2019 [20]. Of note, these samples were collected in a production vineyard where the vines are pruned extensively each year, which is a practice that can have positive effects on grapevine recovery from bois noir disease [42].

Analysis of the RNA-Seq data produced an average of 82,335,298 reads per sample that were aligned to the grapevine reference genome. Out of the 42,413 annotated genes, 24,279 were removed by filtering out those with raw counts over 50 in less than four samples (i.e., genes that were not expressed under any of the conditions). From the remaining 18,134 genes, 15,319 genes (correspond to 84% of the 18,134 expressed genes, and 36% of the 42,413 annotated genes) were significantly differentially expressed in at least one of the comparisons (Table S1).

sRNA-Seq revealed 178 miRNAs (Table S2) and 261 phasiRNAs (Table S3) with raw counts over 50 in at least four samples. Of these, 238 (54%) were significantly differentially expressed. Eighteen of the miRNAs were novel (18 novel MIR loci) and might be involved in novel miRNA-mRNA interactions. Using our pipelines, the novel miRNAs were grouped into 17 novel miRNA families, with two of the loci grouped into the same family, miR14 (Table S4). The prevailing number of differentially expressed sRNAs in the infected grapevines (i.e., 125 sRNAs) in comparison with the number of differentially expressed sRNAs in uninfected plants (i.e., 54 sRNAs) suggests an important role for sRNAs in grapevine responses to infection with ‘*Ca*. P. solani’.

**Figure 1:**
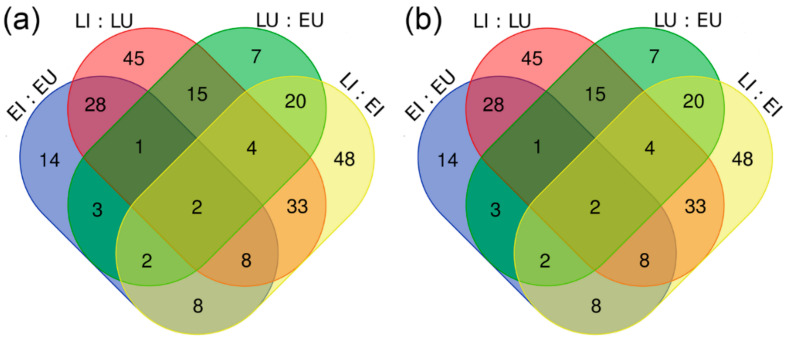
Venn diagrams showing the significantly differentially expressed genes (**a**) and sRNA (**b**) from the 2017 experiment. E, early growing season; L, late growing season; U, uninfected; I, infected with ‘*Ca*. P. solani’.

Venn diagrams reveal some interesting comparisons (Figure 1). Gene expression obtained by RNA-Seq was initially compared between the infected and uninfected grapevine samples (Figure 1a). A total of 6942 and 6288 genes were significantly differentially expressed in infected grapevines compared to uninfected grapevines in the early and late growing seasons, respectively. Together with the same average absolute log_2_ value of fold-change in both cases (i.e., 0.70), these data indicate that the grapevine transcriptional response to infection with ‘*Ca*. P. solani’ in the early season is similar to that of the late growing season. For the differentially expressed genes, 35% (3438 genes) were expressed differentially in both growing seasons, while the remaining 6354 genes were expressed differentially, as either in the early (3504 genes) or the late (2850 genes) growing season.

In the next step, gene expression was compared between grapevine samples from the late and early growing seasons (Figure 1a). In total, 10,941 and 10,998 genes were significantly differentially expressed in the late growing season compared to the early growing season in the uninfected and infected grapevine samples, respectively. Together with the same average absolute log_2_ value of fold-change in both cases (i.e., 1.03), these data suggest that the infection itself did not affect the number of differentially expressed genes during the growing seasons. Moreover, 60% (8187) of the genes were expressed differentially in the uninfected and infected grapevines, while the other 40% (5565) of the genes were expressed differentially either in uninfected (2754 genes) or infected (2811 genes) grapevines.

In contrast with the gene expression revealed, the Venn diagram of sRNAs shows a different picture (Figure 1b). In infected grapevines compared to uninfected grapevines, 66 and 136 sRNAs were differentially expressed in the early or late growing season, respectively. Thirty-nine sRNAs (19%) were expressed differentially in both growing seasons, while the other 163 sRNAs (81%) were expressed differentially either in the early (27 sRNAs) or the late (97 sRNAs) growing season.

Most phytoplasma studies have been focused on the symptomatic phases of pathogenesis, and the consequent conclusions for the more metabolically dynamic late growing season phase are based on the associated results [3,43]. In this regard, the results of the present study are striking, as they show that the asymptomatic early phase of the annual development of bois noir disease is very active at the grapevine transcriptional level, whereas its regulation at the level of sRNAs is more pronounced later on.

**Figure 2:**
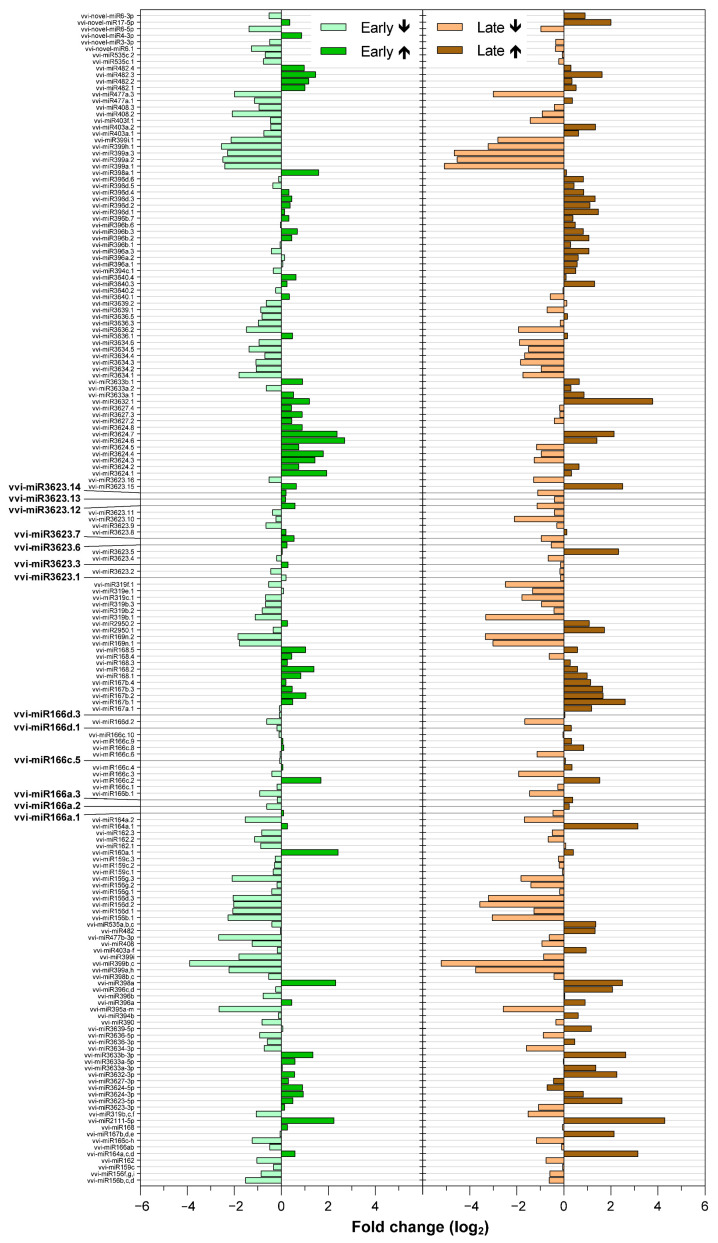
All of the differentially expressed miRNAs in grapevines infected with ‘*Ca*. P. solani’ in comparison with uninfected grapevine during the growing seasons. Exposed are isomiRs of miRNAs, which regulate genes coding for proteins involved in disease resistance with changing mode of up- or down-regulation during the year. ↑, up-regulated; ↓, down-regulated.

Analyzed miRNA and phasiRNA targets have confirmed the growing evidence that any single miRNA might regulate many genes, as well as that any single gene might be targeted by a number of miRNAs (Figure 2; Table S2). An interesting observation was that 57% and 53% of the miRNAs detected were down-regulated in grapevines infected with ‘*Ca*. P. solani’ in the early and late growing seasons, respectively (Figure 2). The significance of this finding is not known at the moment. However, in animals, a global down-regulation of miRNA expression is often the rule in cancers [44]. While 80% of the same miRNAs were similarly differentially expressed either early or late in the growing season, 20% of all miRNAs changed their mode of up-regulation or down-regulation through the year (Figure 2). Among these, there are the most prominent isomiRs with sequences that varied with respect to the reference sequences of *vvi-miR166* and *vvi-miR3623* that regulate genes that encode proteins involved in disease resistance [45].

### 2.2. Gene Set Enrichment Analysis Confirms High Transcriptional Activity in the Early Growing Season

**Figure 3:**
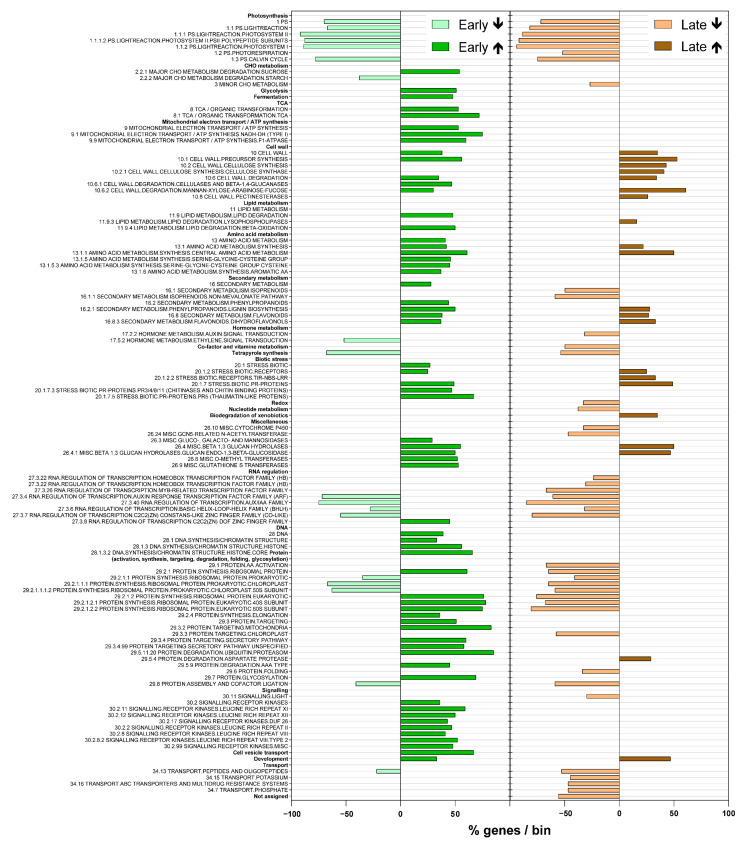
Enriched bins [46] according to gene set enrichment analysis showing proportions (%) of genes up-regulated (↑) or down-regulated (↓) in the particular bins in the grapevines infected with ‘*Ca*. P. solani’ compared to the uninfected grapevines.

Gene set enrichment analysis is a computational method that determines whether an a priori defined set of genes shows statistically significant concordant differences between two biological states. Here it was used to identify any relationships between the expression and function of the differentially expressed genes in these grapevines infected with ‘*Ca*. P. solani’ (Figure 3). This gene set enrichment analysis showed a very strong response of grapevine to infection with ‘*Ca*. P. solani’ early in the growing season, before symptoms develop. Eight functional bins [46] were differentially up-regulated only in the early growing season, specifically as: major CHO metabolism.degradation; glycolysis; fermentation; TCA/organic transformation; mitochondrial electron transport/ATP synthesis; DNA synthesis/chromatin structure; signaling receptor kinases, and cell vesicle transport. Twelve bins were enriched early and late in the growing season, and six bins only late in the growing season (Figure 3).

The largest enriched bins (in terms of percentages of genes) were associated with protein amino acid activation, synthesis of ribosomal proteins, synthesis initiation, elongation, targeting, degradation, folding, glycosylation, and assembly and cofactor ligation. Early in the growing season, the majority of the genes in these bins were up-regulated. Late in the growing season, the majority of the genes in these bins were down-regulated.

The bins related to RNA and transcription factors (i.e., ARF, bHLH, C2C2(Zn) constans-like zinc finger families) were down-regulated for both growing seasons. On the other hand, several genes in the bins associated with the cell wall and secondary metabolism were up-regulated throughout growing seasons.

### 2.3. Genes and miRNAs Not Associated with Phytoplasma Diseases Contribute to Resolving the Sanitary Status of Grapevines

**Figure 4:**
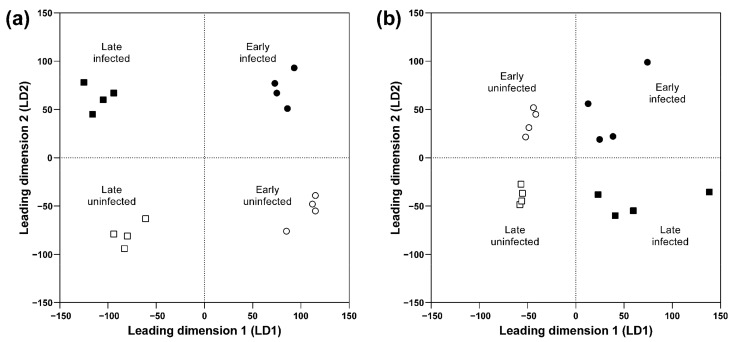
Multidimensional scaling for the normalized gene expression (**a**) and sRNA expression (**b**) in grapevine during infection with ‘*Ca*. P. solani’.

Based on their mRNA profiles, uninfected and infected grapevine samples were clearly separated according to the sanitary status, as well as by the time of sampling (Figure 4a). In addition to the genes that encode a thaumatin protein from pathogenesis-related protein from class 5 and pathogenesis-related protein from class 10 (which have been associated with phytoplasma pathogenesis [3,27]), the other main genes with seasonal variations in these grapevine samples were ones that still have unclear roles during grapevine infection with ‘*Ca*. P. solani’; namely, genes that encode metallothionein, cysteine proteinase1, cellulose synthase-like G3, chloroplast β-amylase, and a gene related to cold, circadian rhythm, and RNA binding2 (Table 1).

The genes that best separated the grapevine samples according to their sanitary status encode metallothionein, which is different from the contributor to the seasonal origin, and the same genes that contribute to separation by season and encode chloroplast β-amylase (i.e., *Vitvi02g00605*) and a protein from the ubiquitin-protein family. Additional contributors that separated the infected and uninfected grapevines are involved in photosynthesis, as the main known process to be down-regulated in phytoplasma-infected grapevines; i.e., the gene that encodes ribulose bisphosphate carboxylase (small chain) family protein, and two genes that encode rubisco activase.

It is worth noting that the Arabidopsis ortholog of chloroplast β-amylase gene *Vitvi02g00605*, *BAM3* (*At4g17090*) encodes one of the six plastidic β-amylases in Arabidopsis, which is transcriptionally induced by cold stress and is dominantly active in mesophyll cells during the night. This might explain the contribution of a gene related to the circadian rhythm to the separation by seasonal origin. The importance of β-amylase to multidimensional scaling might at least partially explain the synthesis and accumulation of starch during phytoplasma infections [9,14,19]. Although one of the prominent symptoms of phytoplasma infections is an accumulation of starch in leaves [9,11,12], the source of this starch is not clear. ADP-glucose-pyrophosphorylase (AGPase) is a rate-limiting enzyme in starch biosynthesis [47]. Hitherto, transcript analysis of the gene that encodes its large regulatory subunit in grapevine cv. Chardonnay when infected with ‘*Ca*. P. solani’ revealed its transcriptional up-regulation [20], but no significant difference on AGPase enzyme activity [19]. Our RNA-Seq of grapevine cv. Zweigelt here showed several genes that encode AGPase, with differential expression in the uninfected and infected grapevines for both growing seasons (Table S1). However, non-significant differences were found in total AGPase enzyme activity (data not shown) in agreement with our previous study [19]. On the other hand, a transcript that encodes chloroplastic β-amylase was up-regulated in infected grapevines in the early growing season and less so in the late growing season (Table 1). This is in agreement with a study on phytoplasma-infected symptomatic mulberry leaves in which the transcript levels of the β-amylase gene were lower compared to uninfected leaves together with a significant reduction in the corresponding β-amylase enzyme activity [12]. These results suggested that the accumulation of starch in the infected leaves results from reduced starch degradation and not from its de-novo synthesis [12]. However, data for β-amylase in mulberry leaves are in agreement with the reduced gene expression and enzyme activity of α-amylase [12]. This was not the case in grapevines of cv. Chardonnay infected with ‘*Ca*. P. solani’, where the infection resulted in increased expression of an α-amylase gene [8]. In grapevine cv. Zweigelt (this study), infection with this pathogen was associated with down-regulation of *Vitvi03g00500* and *Vitvi01g00932*, which are genes that encode α-amylase-like and α-amylase protein, respectively. An additional two genes that encode α-amylase-like proteins, *Vitvi03g01571* and *Vitvi18g00144,* were up-regulated. Whether these data and the previous data on amylases in infected plants are species-related or related to ecological factors is currently not known.

miRNA expression data were also plotted with multidimensional scaling, which clearly revealed the sanitary status of the grapevine samples as well as their early or late growing season (Figure 4b). Interestingly, among sRNAs that differentiated the grapevine samples by sanitary status, there were miRNAs (*vvi-miR482.4*, *vvi-miR166d.2*, *vvi-miR482*, and *vvi-miR156g.1*) that regulated the expression of genes involved in biotic stress, several disease resistance proteins, a heat shock protein, and β-galactosidase (Table 2; Tables S2 and S5). Four sRNAs (*vvi-miR166c-h*, *vvi-miR3623.5*, *vvi-miR3623-5p*, and *vvi-miR3624-3p*) contributed significantly to both sanitary status and season differentiation. These mainly regulate the expression of genes involved in RNA regulation of transcription, protein degradation, and metal transport (Table 2; Tables S2 and S5). The contributors to sanitary status separation also included *vvi-miR162*, *vvi-miR162.3*, *vvi-miR159c*, *vvi-miR159c.1,* and *vvi-miR3623.4*, which regulate the expression of genes involved in nucleotide metabolism, RNA processing and degradation, RNA regulation of transcription, posttranslational protein modification, and protein degradation (Table 2; Tables S2 and S5).

### 2.4. The Most Differentially Expressed Genes and sRNAs Are Associated with Different Aspects of Biotic Stress Signaling

Analysis of the mRNA-Seq and sRNA-Seq data from grapevine cv. Zweigelt during infection with ‘*Ca*. P. solani’ using the MapMan tool [46] revealed several novel patterns of gene expression related to the bins that were putatively related to biotic stress. These included: cell wall; hormones; proteolysis; NBS-LRR receptors; pathogenesis-related proteins; signaling, including sugar and nutrient physiology, receptor kinases, calcium signaling, G-proteins, MAP kinases, and light; transcription factors; and secondary metabolites.

In the bins that included the pathogenesis-related proteins and secondary metabolites, the analysis of the corresponding gene expression in pre-symptomatic but infected plants revealed several genes that were highly up-regulated compared to uninfected grapevines, although their expression decreased late in the growing season (Table S1). Among these, there were genes that encode for pathogenesis-related proteins from class 1 and 5, chitinases, and *Vitvi16g01336*, which encodes 2-oxoglutarate (2OG) and Fe(II)-dependent oxygenase superfamily protein downy mildew resistance 6 (DMR6) ([43,48]. DMR6 has an essential role in the mediation of salicylic acid homeostasis during plant development, leaf senescence, and pathogen responses and acts as a susceptibility S gene in a class of suppressors of plant immunity [27].

**Figure 5:**
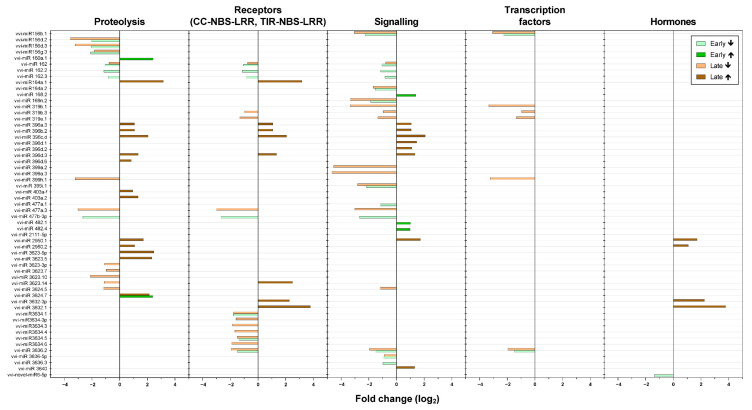
Differential expression of miRNAs associated with biotic stress in the grapevines infected with ‘*Ca*. P. solani’ compared to the uninfected grapevines over the growing seasons. ↑, up-regulated; ↓, down-regulated.

**Figure 6:**
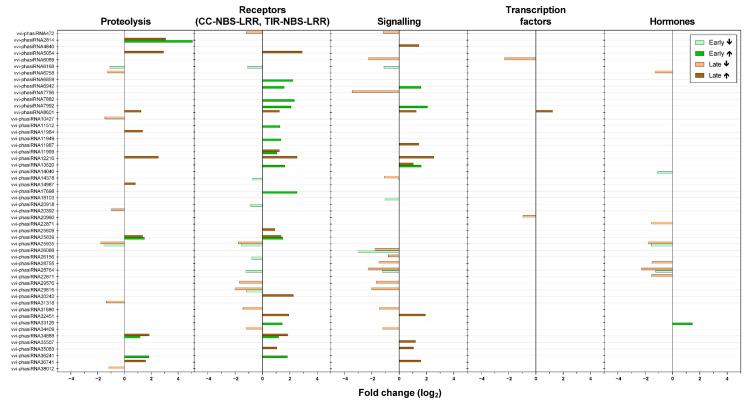
Differential expression of the phasiRNAs associated with biotic stress in the grapevines infected with ‘*Ca*. P. solani’ compared to uninfected grapevines over the growing seasons. ↑, up-regulated; ↓, down-regulated.

In agreement with the reported roles of sRNAs in stresses, the greatest number of predicted targets of differentially expressed miRNAs and phasiRNAs corresponded to the bins associated with the biotic stress signaling (Figure 5 and Figure 6). In this group of sRNAs, 65% of the miRNAs were down-regulated in comparison with the up-regulated miRNAs in both of the growing seasons.

#### 2.4.1. Important Involvement of Genes Associated with the Cell Wall in Bois Noir Pathogenesis

The *cell wall* bin is putatively associated with biotic stress and comprised 262 differentially expressed genes. Early in the growing season, the second most up-regulated gene with an almost 8-fold increase was *Vitvi02g00653*, which encodes expansin-like B1 (Table S1) and is involved in cell wall processes [46]. On the other hand, a gene with the ID *Vitvi09g00767* that encodes expansin B2 was down-regulated (Table S1) and targeted by *vvi-miR166d.2* (Table S5). This miRNA was among those, which differentiated the samples by their sanitary status (Table 2). In the same *cell wall* bin was also the most down-regulated gene of all detected differentially expressed genes with a 9-fold decrease. This was the gene with the ID *Vitvi13g00172*, which encodes expansin A8 and was expressed in the late growing season (Table S1). Although the functional roles of the expansin-like A and B family members remain unclear, a recent study of the expansin-like B1 ortholog in *Brassica rapa* [49] suggested their involvement in stress. In addition, the second most down-regulated gene (log_2_ FC = −6.42) in the early growing season was from the same bin and encodes cellulose synthase-like G3 (*Vitvi02g00165*) (Table S1).

#### 2.4.2. Hormonal Balance Is Disturbed Already in Pre-Symptomatic Phase of Phytoplasma Infection

Infection of grapevine cv. Zweigelt with ‘*Ca*. P. solani’ confirmed the importance of hormonal regulation in phytoplasma diseases [27].

Salicylic acid studies have revealed that several salicylate biosynthetic, signaling, or marker genes are up-regulated in leaf-vein-enriched samples and whole leaves of grapevines infected with phytoplasma ‘*Ca*. P. solani’ [27]. Infection with this phytoplasma for grapevine cv. Zweigelt here showed high induction of three transcripts of several genes that encode for S-adenosyl-L-methionine: salicylic acid carboxyl methyltransferase, which catalyzes the formation of the volatile ester methyl salicylate from salicylic acid (*Vitvi04g02117*, *Vitvi04g02118*, *Vitvi04g02122*); these showed a peak in the early growing season (Table S1). In agreement with the results from a study of grapevine cv. Chardonnay infected with ‘*Ca*. P. solani’ [50], the relative expression of non-expressor of pathogenesis-related protein 1 (*NPR1)*, the receptor for salicylic acid, did not differ across the infected grapevines of this cv. Zweigelt (Table S1). Among the-several genes that encode pathogenesis-related proteins, PR-1, PR-2, and PR-5 are induced by salicylic acid and are commonly used as molecular markers for the salicylic-acid-dependent systemic acquired resistance signaling; they have also been shown to be induced after phytoplasma infection [27]. Out of 14 genes that encode PR-1, the transcription of 12 was induced here in grapevines cv. Zweigelt infected with ‘*Ca*. P. solani’ (Table S1). The same up-regulation pattern was shown for 19 genes that encode PR-5 and also for one gene that encodes PR-2 (Table S1). The *DMR6* gene [27] was also highly induced in the infected grapevine cv. Zweigelt. Similarly, as shown in grapevines infected with Flavescence dorée phytoplasma [14], *DMR6* expression was higher for the early growing season compared to the late season (Table S1).

Several genes that encode the major enzymes involved in jasmonate biosynthesis and modification were differentially expressed (Table S1). Their involvement in phytoplasma infections has been shown for grapevines infected with ‘*Ca*. P. solani’ before, although their role in pathogenicity is not clear yet [27]. In general, genes that encode lipoxygenases are up-regulated upon infection with ‘*Ca*. P. solani’, and they are suppressed upon infection with Flavescence dorée phytoplasma [43]. Lipoxygenase genes have also been shown as down-regulated in Arabidopsis infected with ‘*Ca*. P. asteri’ strain witches’ broom as a result of secreted effector protein11 (SAP11_AYWB_), which destabilizes transcription factor promoting the expression of these genes [51]. A homolog of SAP11_AYWB_ has also been found in the genome of ‘*Ca*. P. solani’ strain SA-1 originally infecting grapevine [52]. However, our RNA-Seq here revealed three genes that encode lipoxygenases (*Vitvi14g00234*, *Vitvi14g02539*, and *Vitvi06g00149*) that were either unaffected or were down-regulated. On the other hand, the transcripts of the genes *Vitvi06g00155* and *Vitvi06g00158* increased from the beginning to the end of the growing seasons, with the same shown for genes that encode the PLAT/LH2 domain of plant lipoxygenase-related proteins (*Vitvi01g01562*, *Vitvi05g00472*, *Vitvi09g00096*, *Vitvi01g01562*, and *Vitvi05g00472*). Similar to a previous study [50], the allene oxide synthase gene (*Vitvi03g00395*) was up-regulated at both sampling times, although a transcript of *Vitvi18g00886* was up-regulated only later in the growing season. In contradiction with the previous reports [50], gene expression of jasmonic acid carboxyl methyltransferases (*Vitvi18g02762*, *Vitvi18g02763*, and *Vitvi18g02761*) was suppressed in infected grapevines. The opposite grapevine response when infected with ‘*Ca*. P. solani’ or Flavescence dorée phytoplasma was additionally supported by induction of gene expression of several 12-oxophytodienoate reductase genes (*Vitvi18g02485*, *Vitvi18g02138*, *Vitvi18g02139*, *Vitvi18g03161*, and *Vitvi18g03162*) early in the growing season in grapevine cv. Zweigelt infected with ‘*Ca*. P. solani’. Gene expression of jasmonic acid ZIM (zinc-finger inflorescence meristem) domain-containing protein, which was shown to be down-regulated upon infection with Flavescence dorée phytoplasma, in grapevine cv. Zweigelt depended here on the specific gene: while *Vitvi09g00064* and *Vitvi01g02293* were up-regulated in both growing seasons, *Vitvi01g00473* and *Vitvi17g00189* were down-regulated, and *Vitvi10g01879* was down-regulated in the early growing season and up-regulated in the late season. The PR3/4 marker genes for jasmonic acid metabolism were up-regulated only in grapevines that recovered from the infection of grapevine cv. Chardonnay with ‘*Ca*. P. solani’ [50]. On the other hand, in grapevine cv. Zweigelt, four of the genes that encode PR3/4 (*Vitvi04g01049*, *Vitvi05g00094*, *Vitvi05g01366*, and *Vitvi05g01575*) were not affected, while the transcripts of *Vitvi05g02250*, *Vitvi15g01035,* and *Vitvi15g01037* (which also encode PR3/4) increased, especially during the early growing season. The transcript levels of a third jasmonic acid metabolism marker PR6 (*Vitvi02g01273*, *Vitvi05g01910*, *Vitvi18g00852*, *Vitvi18g03048*, *Vitvi09g00071*, *Vitvi11g00061*, *Vitvi17g01613*, and *Vitvi17g01121*) were higher later in the growing season compared to the early season. The families of jasmonic acid metabolism genes revealed here included at least one gene for which expression has been shown previously, which indicates the importance of high-throughput analysis for interpretation of the not always straightforward functions in infected plants.

The role of auxins in phytoplasma infections has already been documented [27], and the present data supported this, with differential expression of more than 200 auxin-associated genes (Table S1). *Small auxin-up RNAs* (*SAURs*) comprise a large multigene family that is involved in primary auxin responses in plants. These influence nearly all aspects of plant growth and development through the regulation of cell division, expansion, differentiation, and patterning. However, the functions of the SAUR proteins have remained elusive, presumably due to extensive genetic redundancy [53]. The present analysis revealed four down-regulated SAUR genes (*Vitvi02g00507*, *Vitvi03g01350*, *Vitvi04g02073*, and *Vitvi09g00046*). This is in agreement with a study of a single virulence factor, tengu-su inducer (TENGU), that is associated with the phytoplasma-infected plant phenotype [54,55]. Among the genes that directly influence the homeostasis of auxins, there is the auxin-responsive GH3 gene family. The related *Vitvi03g00586*, *Vitvi03g00586,* and *Vitvi07g01644* genes were up-regulated in the infected grapevines of cv. Zweigelt. To date, their role in phytoplasma infections has not been evaluated.

Fifty-nine of the genes revealed as associated with ethylene metabolism were affected by infection of grapevine cv. Zweigelt (Table S1). Among these, four were up-regulated for a more than 3-fold difference in the early growing season: *Vitvi07g02070,* which encodes ERF098; Vitvi08g01502, which encodes a transmembrane protein; and *Vitvi09g00834* and *Vitvi09g00837*, which encode two integrase-type DNA-binding superfamily proteins. *Vitvi08g01502* was also induced later in the growing season, together with *Vitvi04g00533*, which is also an integrase-type DNA-binding superfamily protein. On the other hand, two genes from the ethylene signaling bin were down-regulated later in the growing season, with a log_2_ FC greater than 3: *Vitvi05g01924*, which encodes a 2-oxoglutarate (2OG) and Fe(II)-dependent oxygenase superfamily protein; and *Vitvi04g01895*, which encodes a PPPDE  (permuted papain fold peptidases of DsRNA viruses and eukaryotes) thiol peptidase family protein. The significance of the expression of these genes for phytoplasma pathogenicity has not been explored.

Although less studied, plant hormones such as abscisic acid, gibberellic acid, cytokinins, brassinosteroids, and peptide hormones have important roles in plant defense against invading organisms through their fine-tuning of the plant responses to phytoplasmas [27].

In the early growing season, more than 3-fold increases were seen for gibberellin 3-oxidase 1 (*Vitvi04g00435*) and a proline-rich protein (*Vitvi14g01819*). In the later growing season, an overall decrease in gibberellin oxidase gene expression was seen (*Vitvi10g00020, Vitvi19g00432, Vitvi15g00782, Vitvi09g00448,* and *Vitvi17g00601*).

Although 45 genes associated with abscisic acid metabolism were affected by ‘*Ca*. P. solani’ infection (Table S1), their differential expression did not exceed four-fold change, with the exceptions of the genes *Vitvi03g01727* and *Vitvi12g00015*, which encode HVA22 protein; these were shown to be involved in abiotic stress in *Citrus* spp. [56].

For brassinosteroids, high down-regulation in the late growing season was observed for two members, both of which are involved in sterol synthesis (*Vitvi19g00007* and *Vitvi01g00319*).

We also detected changes in both miRNAs and phasiRNAs associated with predicted targets in hormone metabolism (Figure 5 and Figure 6). Early in the growing season, one sRNA associated with genes related to jasmonic acid was up-regulated (*vvi-phasiRNA33126*), while *vvi-phasiRNA33126* was down-regulated. On the other hand, *vvi-phasiRNA25935* and *vvi-phasiRNA28764,* which are associated with auxins, and *vvi-novel-miR6-5p,* which is associated with abscisic acid, were down-regulated. Early in the growing season, there were no differentially expressed sRNAs associated with brassinosteroids or ethylene. While 71% of detected sRNAs that targeted genes in the bin of auxins were down-regulated late in the growing season (*vvi-phasiRNA25935*, *vvi-phasiRNA*28755*, vvi-phasiRNA6258,* and *vvi-phasiRNA22871*), two miRNAs were up-regulated (*vvi-miR2950.1* and *vvi-miR2950.2*). Later in the growing season, there was down-regulation of *vvi-miR3624.5* and *vvi-phasiRNA31318*, which are associated with brassinosteroids and ethylene, respectively. In addition, two miRNAs that are associated with jasmonic acid were up-regulated (*vvi-miR3632-3p* and *vvi-miR3632.1*), while *vvi-phasiRNA14040* was down-regulated.

### 2.5. Known Pathways with the Involvement of Novel Genes

As photosynthesis and secondary metabolism are tightly intertwined with symptom development in the later growing season [3,43], they have been most studied in plants infected with phytoplasmas. However, their role in pre-symptomatic plants has been less considered. Of note, phasiRNAs were up-regulated in the bins associated with photosynthesis (Table S3), namely vvi-phasiRNA2814 in PS.lightreaction.photosystemII.PSII polypeptide subunits in the early and late growing season, as well as vvi-pahsiRNA12216 in PS.calvincycle.rubiscointeracting later in the growing season. These findings appear to be related to significant decreases in gene transcripts from the same bins.

**Figure 7:**
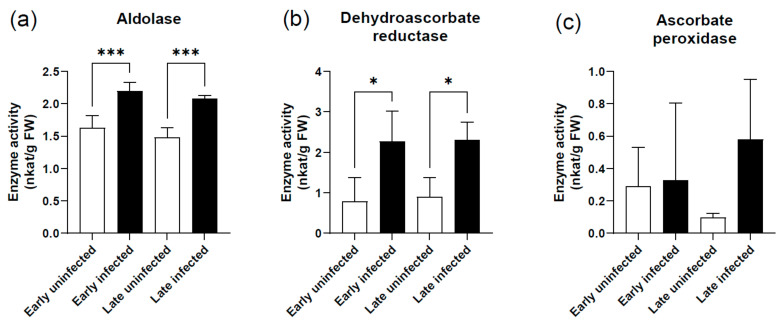
Enzyme activities of (**a**) aldolase, (**b**) dehydroascorbate, and (**c**) ascorbate peroxidase for the grapevine cv. Zweigelt. *, *p* <0.05; ***, *p* < 0.001 (one-way ANOVA with Tukey’s post-tests).

Glycolysis and oxidative stress processes were also affected in grapevine cv. Zweigelt infected with phytoplasmas, with up-regulation of several aldolase genes that accelerate the reversible conversion of fructose-1,6-bisphosphate to dihydroxyacetone-phosphate and glyceraldehyde-3-phosphate (*Vitvi19g01724*, *Vitvi01g00360*, and *Vitvi08g01506*) (Table 1), and this correlated with detection of the activity increase of their encoded enzymes (Figure 7). The transcript of *Vitvi13g00241* that encodes dehydroascorbate reductase showed a small increase in the late growing season in response to infection (Table S1), but its enzymatic activity was significantly higher for both the early and late sampling times (Figure 7), indicating a possible posttranslational regulation. Lastly here, the expression of seven ascorbate peroxidase genes (*Vitvi04g02166*, *Vitvi06g00358*, *Vitvi18g00256*, *Vitvi08g01143*, *Vitvi03g00137*, *Vitvi04g00484*, and *Vitvi18g00445*) (Table S1) were differentially regulated in response to infection, likely contributing to a statistically insignificant difference in total ascorbate peroxidase activity in grapevine cv. Zweigelt (Figure 7). A role for ascorbate peroxidase in grapevine phytoplasma infections has been shown before, including in grapevines recovered of disease [57,58,59,60,61].

Previous studies have shown that phytoplasma infections affect carbohydrate metabolism [14,19,43]. Expression profiling analysis of the infected samples in the present study revealed several genes that encode starch synthase (Table S1). Three of these (*Vitvi10g02394*, *Vitvi10g00094*, and *Vitvi14g01968*) were down-regulated early in the growing season with small increases in the transcript levels later; two others (*Vitvi10g00739* and *Vitvi11g00903*) were down-regulated at both sampling times. As we have shown previously [14,19], sucrose synthase gene expression was higher in infected grapevines (*Vitvi04g00831*, *Vitvi07g00353*, *Vitvi11g00030*, and *Vitvi17g01221*), which indicated the important role of this enzyme in phytoplasma pathogenicity.

In agreement with the symptom development of phytoplasma-infected grapevines, the secondary metabolism changes are also pronounced. Previously observed changes in flavonoid synthesis genes [3,43] were also noticeable here for grapevine cv. Zweigelt. However, comparisons of the responses in the early and late growing seasons showed higher up-regulation in the early growing season for anthocyanins, chalcones, and dihydroflavonols, while isoprenoids were more commonly up-regulated in the late growing season (Table 1).

Abnormal lignification of canes is a prominent symptom of bois noir disease [62], and accordingly, several genes related to lignin biosynthesis are affected in these infected grapevines. Among these, there was up-regulation of phenylalanine ammonia-lyase in infected plants, which has also been shown in grapevine cv. Chardonnay infected with the same phytoplasma [26]. Our analysis additionally revealed several down-regulated laccase genes in the late growing season (*Vitvi18g01438*, *Vitvi18g02906*, *Vitvi04g01984*, *Vitvi08g01335*, *Vitvi08g01223*, and *Vitvi08g01031*). The biological functions of a diverse superfamily of multicopper oxidases and laccases include the lignification responsible for maintenance of the cell wall structure and of mechanical rigidity [63].

### 2.6. Temporal Network Modeling Reveals New Cross-Talk between Pathways Involved in Grapevines Infected with ‘Ca. P. solani’

**Figure 8:**
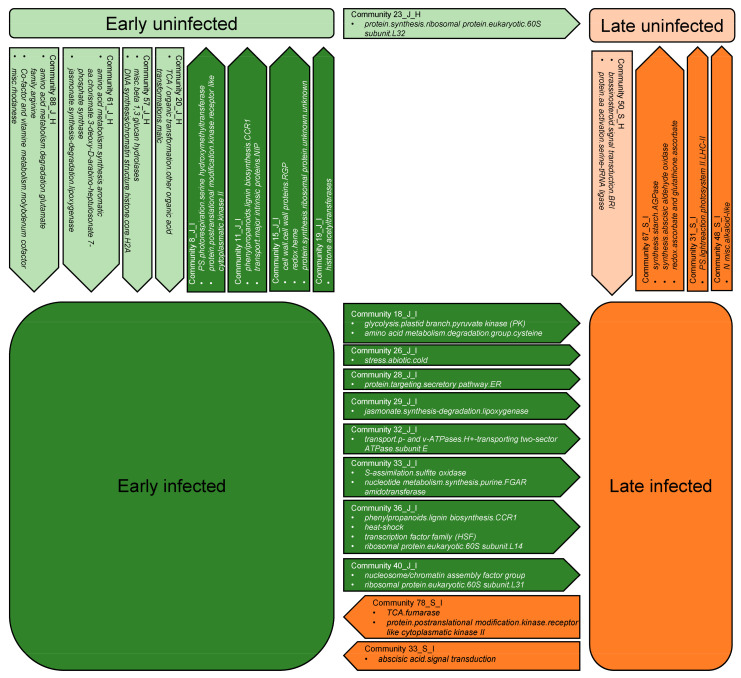
Schematic presentation of the communities with bins [46] that disintegrate between groups of uninfected (U) and grapevines infected (I) with ‘*Ca.* P. solani’ in the early (E) and late (L) growing season.

A drawback of most transcriptomic studies on phytoplasma-infected grapevines growing in their natural environment is that sampling occurs only at one time point in the growing season, which is most often when disease symptoms are most pronounced [3]. Only a few studies have been conducted in the early and late growing seasons, which have resulted in a short list of genes and their protein products; some of these have been explored in more detail [14,26,50]. Using a novel integrated analysis of transcriptomic data known as network enrichment methodology, which is based directly on RNA-Seq data [39], we explored the grouping of genes in communities that: (i) formed at the same times (i.e., early or late in the growing season) in the same groups of grapevines (i.e., uninfected and infected with ‘*Ca*. P. solani’); and (ii) showed significant disintegration (i.e., separation with a significant dissipation index) between the two growing seasons and between each group of grapevines (Figure 8, Table S6). By analyzing community behavior with time, we discovered several new genes that are involved in phytoplasma pathogenesis and are connected in yet unexplored networks.

#### 2.6.1. Disintegrated Communities in Infected Grapevines

In the early growing season, in the group of uninfected grapevines, we detected four communities of genes that disintegrated with a high dissipation index in infected grapevines (Figure 8, Table S7). The first community consisted of genes functionally classified as β-1,3-glucan-hydrolases and H2A histone core proteins, which are involved in DNA synthesis. The latter included up-regulated plasmodesmata callose-binding protein 3 (*Vitvi01g00051*) in the infected grapevines in agreement with previous studies where callose was shown to be involved in phytoplasma pathogenicity [8,64,65].

The second community consisted of genes functionally classified as malic acid transformation enzymes, which included two induced genes in infected grapevines, the MATE (multidrug and toxic compound extrusion) efflux family protein (*Vitvi12g00101*), and the calcium-binding EF-hand family protein (*Vitvi14g00470*). Multidrug and toxic compound extrusion transporters are one of the largest secondary active transporter families in plants. These are involved in a wide variety of physiological functions throughout plant development for the transport of a broad range of substrates such as organic acids, plant hormones, and secondary metabolites [66,67]. They have been shown to be linked to disease resistance associated with salicylic acid [68], as well as to abscisic acid sensitivity and drought tolerance [69]. Activation of salicylic acid metabolism in phytoplasma-infected grapevines in the early growing season has already been documented [14,50]. On the other hand, the calcium-binding EF-hand proteins have roles in the resistance mechanisms to various biotic and abiotic stresses [70,71].

**Figure 9:**
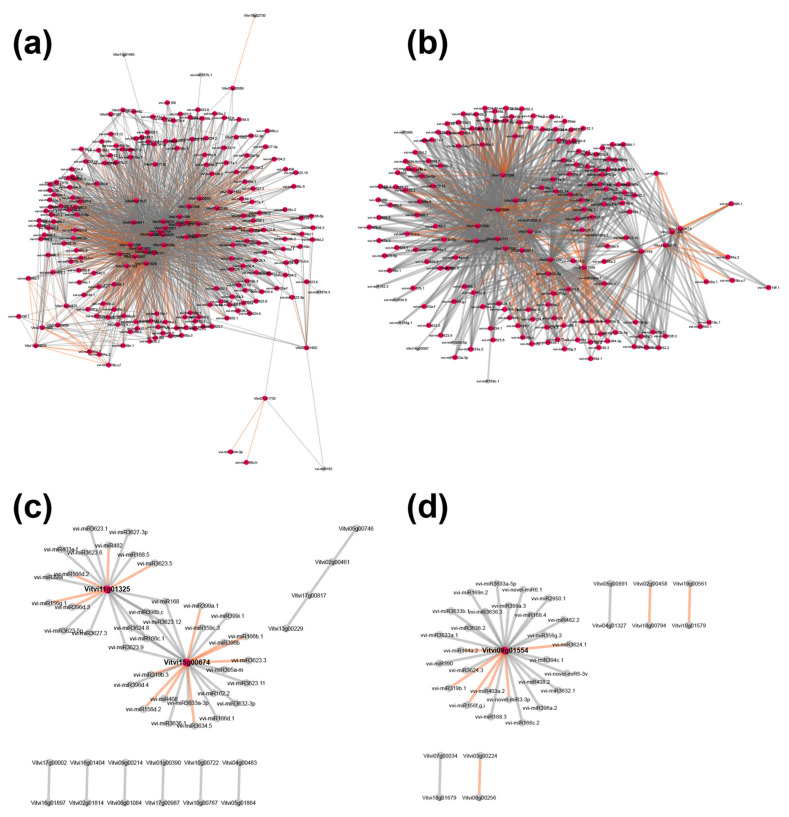
Visualization of networks that included mRNAs and miRNAs with Cytoscape: (**a**) uninfected, early growing season; (**b**) infected with ‘*Ca*. P. solani’, early growing season; (**c**) uninfected, late growing season; (**d**) infected with ‘*Ca*. P. solani’, late growing season.

Functional classification of the genes that comprise the third community consisted of arginine degradation, molybdenum involvement in vitamin metabolism, and rhodanese. Here, a molybdenum cofactor sulfurase (*Vitvi01g00087*) was significantly increased upon infection (Figure 9, Table S1). This enzyme is required for the enzymatic activity of the Mo enzymes (e.g., aldehyde oxidase) that are essential for the biosynthesis of the bioactive compounds abscisic acid and allantoin [72,73]. In Arabidopsis, this enzyme contributes to anthocyanin accumulation and oxidative stress tolerance in abscisic acid-dependent and -independent ways [74]. These processes have also been shown to be associated with phytoplasma infections previously [27,43,75].

The last community contained some genes that encode jasmonic acid lipoxygenases, which were discussed above in Section 2.4.2. (Table S1).

#### 2.6.2. Early Growing Season Communities with a High Dissipation Index in Uninfected Grapevines

It is nevertheless worth comparing the communities that formed early in the growing season in infected grapevines, which disintegrated in uninfected grapevines with a very high dissipation index. With the applied methodology, we detected four such communities (Figure 8, Table S7). A high dissipation index of 0.62 was seen for a community that involved the genes from bins *photosynthesis.photorespiration.serine hydroxymethyltransferase* and *protein.postranslational modification.kinase.receptor-like cytoplasmatic kinase II*. Although photosynthesis is among the most studied processes during phytoplasma infection, in general, the genes revealed in this community have never before been described as responsive to phytoplasma infection. A possible reason for this oversight might be their low fold-changes between the uninfected and infected grapevines.

In the second community, there were two bins: *secondary metabolism.phenylpropanoids.lignin biosynthesis.CCR1* and *transport.major intrinsic proteins.NIP*. It is becoming increasingly evident that secondary metabolism is greatly affected during bois noir disease, which has been shown at the transcriptional, proteome, and metabolome levels [3]. A gene from this community (*Vitvi06g01762*) encodes a UDP-glycosyltransferase superfamily protein and its transcript increased in infected grapevines compared to uninfected grapevines (Table S6). It is known that glycosylation with UDP-glycosyltransferases is a major regulator of phenylpropanoid availability and biological activity in plants [76], and this protein family indicates stress-responsive regulation in Arabidopsis and *Brassica* species [77]. Although reports of cytokinin involvement in phytoplasma–plant interactions are scarce and mainly indirect, some symptoms of phytoplasma-infected plants hint at cytokinin participation (e.g., witches’ broom, leaf yellowing, and fasciations) [27]. Thus, the observed induced transcript of CycD3 might be related to plants with phytoplasma infection phenotypes.

In the community with a dissipation index of 0.66 that was composed of the bins *cell wall.cell wall proteins.RGP*, *redox.heme,* and *protein.synthesis.ribosomal protein.unknown.unknown*, there was prominent differential expression of *Vitvi14g02435*, a gene that encodes a germin-like protein 10. This protein has been induced in soybean by methyl jasmonate, ethylene, and salicylic acid [78], which are all involved in defense responses and in phytoplasma infections [27]. In addition, overexpression of germin-like protein 10 in transgenic tobacco significantly enhanced tolerance to *Sclerotiorum* infection [78].

#### 2.6.3. Early versus Late Growing Season Communities in Infected Grapevines

A comparison of communities that disintegrated from the early growing season toward the late growing season, and vice versa, showed eight communities with a high dissipation index in the former and only two in the latter group (Figure 8, Table S7). However, communities in the first group included genes with a wide array of functional classifications. This finding suggests a large transcriptional dynamic prior to symptom development, which previously used methods failed to capture.

#### 2.6.4. Exploring mRNA-mRNA-miRNA Interaction Networks

We extended the idea of analyzing mRNA-mRNA expression similarity networks to also include miRNA molecules for each time and grapevine state point, which resulted in four individual networks. During community detection, the majority of the miRNA molecules were pruned due to low correlation values, which resulted from large expression differences between mRNA and miRNA. To remedy this situation, a novel type of connection between miRNA and mRNA molecules was introduced, based on the shortest path searches.

Following this approach, the complex network of connections both in uninfected and infected grapevines early in the growing season were defined (Figure 9). In particular, we detected only a few connections late in the growing season in uninfected grapevines, and even fewer in the infected grapevines, such as the gene with the ID *Vitvi15g00674* that encodes SPX (SYG1/Pho81/XPR1) domain protein 3, which has been shown to have a role in signaling and homeostasis of cellular phosphate [79]. In addition, the transcript of this gene was induced in transgenic plants that expressed the effector SAP11_AYWB_ [80]. It was shown in that study that SPX domain protein 3 is suppressed when SAP11_AYWB_ is expressed under a *phosphate starvation response1* (*phr1*) mutant background, which suggested that *PHR1* is required for SAP11_AYWB_-triggered cellular phosphate starvation responses. Of note, *PHR1* encodes the MYB (myeloblastosis) transcription factor that has a key role in responding to cellular phosphate deficiency in Arabidopsis [81]. However, the transcript of SPX domain protein 3 was down-regulated upon ‘*Ca*. P. solani’ infection of grapevine cv. Zweigelt, although the transcripts of *PHR1* (*Vitvi14g00736* and *Vitvi07g00666*) were slightly increased. As has been shown before, there is no universal response to phytoplasma infections [3,27,43].

In infected grapevines later in the growing season, several miRNAs were connected to only one gene, namely *Vitvi09g01554* (Figure 9, Table S1). This gene has not been assigned to any bins and is annotated as PTHR36328:SF1, a cryptdin protein-like protein. Its mammal ortholog is an α-defensin known as cryptdin, which is a major microbicidal constituent of Paneth cell granules in mouse intestinal epithelial cells. Cryptdin is actively involved in innate enteric immunity and maintains intestinal homeostasis through the control of the intestinal microbiota [82,83,84]. Although its role in phytoplasma pathogenicity is entirely unknown, it might be a suitable candidate for exploring its presumed similar role in plant defense response against phytoplasmas.

## 3. Conclusions

This is the first comprehensive high-throughput RNA-Seq and sRNA-Seq study of grapevine infected with ‘*Ca*. P. solani’ for two consecutive growing seasons, and it has resulted in several striking findings. We show a very dynamic transcriptional activity in infected grapevines in the early growing season (i.e., prior to symptom development). Grapevine plants reacted to the infection with two distinct sets of genes that responded at each growing phase. This reprogramming was not visible for the sRNA levels during the early time point of infection. However, the number of differentially expressed sRNA significantly increased with the annual development of bois noir disease, the majority of which were associated with biotic stress processes. This study has also revealed the importance of less-studied aspects of hormonal involvement in phytoplasma pathogenicity. The interaction network methodology introduced here enabled us to explore the temporal interaction network dynamics, and hence to discover new mRNAs and sRNAs, which might be crucial for an understanding of bois noir development over the growing seasons and might open new routes for research into phytoplasma diseases.

## 4. Materials and Methods

### 4.1. Plant Material

Four phytoplasma-infected grapevines (*Vitis vinifera*) cv. Zweigelt (syn. Rotburger,) and their four healthy neighbors from a vineyard in Klosterneuburg (Austria) were selected for the study, according to the development of their symptoms in the previous season (2016). The grapevines were planted in 2006, and the rootstock is Kober 5 BB. The first fully developed leaves were sampled from several canes of each vine in June and September 2017 and then in June 2020. The leaf veins were cut out of the leaves sampled, flash-frozen in liquid nitrogen, ground to a fine powder, and stored at −80 °C. In 2017, all four of the selected phytoplasma-infected grapevines showed severe symptoms in September, while three of these four phytoplasma-infected grapevines recovered in 2020.

### 4.2. Detection of Phytoplasma

In September 2017, all four phytoplasma-infected grapevines showed typical symptoms of infection, including reddening and curling of leaves and no or incomplete fruit development. The presence of ‘*Ca*. P. solani’ was confirmed by PCR with *SecY*, *Stamp*, *tuf,* and *vmp1* specific primers as described previously (Aryan et al., 2014). Based on these four markers, the previously described nettle type CPsM4_At1 was detected for all four of the infected grapevines. For the four control grapevines, none of the markers provided any PCR signal. All of the grapevines were free of symptoms of powdery and downy mildew. ESCA symptoms had not been recorded for any of the tested grapevines since 2006. The same grapevines were additionally sampled in 2020 and were again tested for the phytoplasmas in the late growing season by PCR and by visual characterization. However, the presence of phytoplasmas could not be detected in three out of the four infected grapevines, which clearly showed remission of bois noir disease.

### 4.3. RNA Extraction and Sequencing

Total RNAs, including small RNAs, were extracted from vein-enriched leaf samples from each plant separately, using an optimized cetyltrimethylammonium bromide (CTAB) method (adapted from [85]) combined with RNA purification on columns (Zymo-Spin; Direct-zol RNA MiniPrep Plus kits, Zymo Research, Irvine, CA, USA). About 50 mg frozen and powdered tissue was further homogenized with steel beads for 10 min at maximum speed in 800 μL CTAB buffer (100 mM Tris-HCl, pH = 8, 2 M NaCl, 25 mM EDTA, 2.0% (*w/v*) CTAB, 2.5% (*w/v*) PVP40, 2.0% (*v/v*) β-mercaptoethanol) using TissueLyser (Qiagen, Hilden, Germany). After the addition of an equal volume of chloroform-isoamyl alcohol (24:1), the sample was vortexed and centrifuged for 10 min at 10,000× *g* at 4 °C. The upper aqueous phase was recovered, to which 1.5 volume of pure ethanol was added. After a 30 min precipitation at 4 °C, the mixture was transferred into the columns (Zymo-Spin, Direct-zol RNA MiniPrep Plus kits, Zymo Research). The RNA was purified according to the manufacturer’s instructions, with an additional washing step and a second prewashing step added to the beginning of the purification process. To elute the RNA, 30 μL preheated (80 °C) DNase/RNase-free water was added to the column and incubated for 10 min at room temperature. This was followed by 1 min centrifugation at 14,000× *g*. The isolated RNA was subjected to DNase digestion (DNase I Set; Zymo Research) and cleaned up using RNA Clean & Concentrator kits (Zymo Research). RNA concentration, integrity, and purity were assessed using a Bioanalyser (2100) and RNA 6000 Nano kits (Agilent Technologies, Santa Clara, CA, USA). Library preparations for the mRNAs and sRNAs, sequencing services (HiSeq 4000, Illumina, San Diego, CA, USA), and preprocessing to remove the adapter sequences and low-quality reads were provided by Novogene (Hong Kong). The raw data (in fastq format) have been deposited with the European Nucleotide Archive (ENA) under project accession number PRJEB42777. 

### 4.4. mRNA Data Analysis

The 150 bp paired-end reads obtained were trimmed to remove low-quality bases (Phred < 20), clipped to remove the remaining adapter sequences, and mapped to the 12X.2 version of the PN40024 grapevine reference genome (https://urgi.versailles.inra.fr/files/Vini/Vitis%2012X.2%20annotations/, 29 February 2021), using CLC Genomics Workbench 12.0 (Qiagen), with the following parameters: mismatch cost, 2; insertion or deletion cost, 3; length fraction, 1.0; similarity fraction, 0.95; and maximum number of hits for a read, 1. The reads were annotated using the VCost.v2 annotation.

The differential expression analysis was performed in R v3.4.2 [86], using the limma package v3.34.9 [87]. mRNA counts with a baseline expression level of >50 reads mapped in at least 4 samples were TMM-normalized in edgeR v3.20.9 [88] and transformed using voom [89]. To identify differentially expressed mRNAs, the empirical Bayes approach was used, with Benjamini and Hochberg’s (FDR) *p*-value adjustment. Genes with adjusted *p*-values <0.05 were considered statistically significantly differentially expressed.

### 4.5. sRNA Data Analysis

sRNA reads were filtered to exclude reads shorter than 18 nt and longer than 26 nt, as well as reads that were matching to rRNAs, tRNAs, snRNAs, and snoRNAs in the RNACentral database (https://rnacentral.org, 29 February 2021) [90], and using a CLC Genomics Workbench v8 (Qiagen). To identify known grapevine miRNAs, the remaining preprocessed sRNA reads were compared to the grapevine miRNAs registered in the miRBase database, release 22 (http://www.mirbase.org, 29 February 2021) [91], which did not allow mismatches. To identify novel unannotated miRNAs and their loci of origin (i.e., MIR loci), the reads were submitted to the two plant miRNA prediction tools ShortStack and miR-PREFeR [92,93]. Predictions were performed using the default parameters, except that no mismatches were allowed during mapping on reference 12X.2 version of the PN40024 grapevine reference genome (https://urgi.versailles.inra.fr/files/Vini/Vitis%2012X.2%20annotations/, 29 February 2021). MiRNAs were considered novel miRNAs only if they had >5 raw reads in at least two sRNA libraries and the miRNA sequence, and the corresponding miRNA* and MIR locus should have been predicted with both miRNA prediction tools. Reads that mapped to more than 30 locations in the grapevine genome were also discarded as being too repetitive to be miRNAs. As the miRNA prediction tools output also contained predictions of already annotated grapevine MIR loci, annotated grapevine miRNA precursors (pre-miRNA) from miRBase (v22) were mapped to the reference grapevine genome using bowtie2 [94]. Next, genome locations were extracted and compared with the predicted MIR loci using our internally developed script. If no overlap was detected, the predicted MIR loci were regarded as novel MIR loci. Novel grapevine miRNAs were further classified into known or novel miRNA families by clustering their predicted pre-miRNA sequences with sequences of known plant pre-miRNAs from miRBase using CD-HIT-EST, with an identity threshold of 0.8 [95]. The sequences with similarities with annotated pre-miRNAs were grouped into the corresponding known miRNA families, and sequences that did not show similarities with known plant miRNAs were classified as novel miRNA families.

Additionally, sequence miRNA variants (isomiRs) of known and novel miRNAs were identified using isomiRID [96]. Only sRNAs that matched perfectly to known or novel pre-miRNA sequences were considered (i.e., templated isomiRs). Prediction of PHA S (phasiRNA-producing) loci was performed using unitas [97]. The PHA S loci were detected by mapping preprocessed sRNA reads to the grapevine transcriptome sequences (Vitis 12X.2 annotations; https://urgi.versailles.inra.fr/files/Vini/Vitis%2012X.2%20annotations/, 29 February 2021) at 21-nt and 24-nt intervals and default settings.

The preprocessed reads from sRNA-Seq samples were mapped (with no mismatches allowed) to all known grapevine miRNAs, novel miRNAs, and isomiRs and counted using our internally developed script. Raw counts were exported and deposited with the European Nucleotide Archive under the project accession number PRJEB42777. Differential expression analysis was performed in R v3.2.2 [86] using the limma package v3.34.9 [87]. sRNA counts with a baseline expression level of >50 reads mapped in at least four samples were TMM-normalized in edgeR v3.20.9 [88] and transformed using voom [89]. To identify differentially expressed mRNAs, the empirical Bayes approach was used with the Benjamini and Hochberg (FDR) *p*-value adjustment. Genes with adjusted *p*-values <0.05 were considered statistically significantly differentially expressed.

### 4.6. sRNA Target Prediction

In-silico identification of grapevine transcripts targeted by sRNAs was carried out using the psRNATarget [98] and grapevine transcriptome sequences (Vitis 12X.2 annotations; https://urgi.versailles.inra.fr/files/Vini/Vitis%2012X.2%20annotations/, 29 February 2021), with the maximum expectation parameter set to 3 (Expectation), and otherwise using the default parameters.

### 4.7. Differential Expression Analysis and Visualization of mRNA and sRNA Expression

Samples were visualized for the level of their similarity using multidimensional scaling, as calculated from normalized mRNA and sRNA expression values separately. Principal component analysis in R v3.4.2 [86] was used to extract the genes that contributed most to the first two leading dimensions (LD1, LD2). To compare seasonal effects of infections, gene set enrichment analysis [99] was performed on normalized expression values using a grapevine MapMan mapping file [100]. The collapse/remap to gene symbols option was enabled (No_collapse), permutation was set to gene sets, and otherwise, the default settings were used. Functional categories with a false discovery rate corrected q ≤ 0.05 were considered significant.

### 4.8. Network Community Analyses

The similarity between mRNA expression data was calculated as the reciprocal value of similarities between the mRNA expression data were calculated as the reciprocal value of the Euclidean distance for a given pair of expression vectors for each individual time, as the grapevine state points. The application of automated thresholding on these similarity networks allowed easier exploration of the space of possible networks. Scale-free networks (2 > α > 3; α being the exponent of the fitted power-law function) were used for community detection with Infomap [101]. To generate mRNA–miRNA networks, connections were added by using shortest path searches of maximum length 3 [102]. Networks were visualized using Cytoscape [103].

### 4.9. Targeted Grapevine Gene Expression Analysis by qPCR

Differential expression of four genes was confirmed with qPCR (Table S7): for *DMR6* (*Vitvi16g01336*), *OLP* (*Vitvi02g01404*), *SAMT* (*Vitvi04g02122*), and *LOX* (*Vitvi06g00158*), with UBI_CF as the reference (*Vitvi19g00744*). These genes were chosen based on our previous results that showed their involvement in bois noir pathogenesis [8,20]. The complete list of primers and probes used is given in Table S7 (MIQE). Reverse transcription was performed with the High-Capacity RNA-to-cDNA kits (Applied Biosystems, Foster City, CA, USA). FastStart Universal Probe Master (Roche, Penzberg, Germany) was used for qPCR. The following thermal cycle conditions were applied: 95 °C for 10 min, 40 cycles of 95 °C for 15 s, and 60 °C for 1 min for PCR, and a climb in increments of 0.05 °C from 60 °C to 95 °C for the high-resolution melting curve. The Cq values were used for the relative calculation of initial target numbers from a serial dilution curve using quantGenius [104].

### 4.10. Enzymatic Activities

Extraction of enzymes from the grapevine material was performed as previously described by Jammer et al. (2015) [105] and adjusted according to Anžič (2019) [106]. The grapevine material was ground in liquid nitrogen using a mortar and pestle. About 0.5 g of material was collected into 2 mL microcentrifuge tubes (Eppendorf, Germany). Then 1 mL extraction buffer was added (0.5 M MOPS, 5 mM MgCl_2_, 0.5 mg/mL BSA, 0.05% Triton X-100, 25 µM dithiothreitol, 1 mM benzamidine, 3% PEG-4000, 0.1 mM phenylmethylsulphonyl fluoride, 1% *polyvinylpyrrolidone*). The samples were then mixed on a rotary shaker for 40 min at 4 °C, and then they were centrifuged at 20,000× *g* for 10 min at 4 °C. The supernatant contained the cytoplasmic fraction of the enzymes, and it was dialyzed overnight against 20 mM KPO_4_ buffer, pH 7.4. After dialysis, the protein-extract aliquots were pipetted into 96-well plates and stored at −20 °C. All of the enzymatic activity assays were performed in UV-transmissive, flat-bottomed, 96-well plates (UV-Star Greiner BioOne; Kremsmünster, Austria). Protein extract volumes from 1 µL to 20 µL were used for the reactions. The total reaction volume was 160 µL. Reaction mixes were incubated in a plate reader (Ascent Multiskan; Thermo Fisher Scientific, Waltham, MA, USA) for 40 min at 25 °C or 30 °C, according to the optimized protocol for each enzyme. All of the assays were carried out in triplicate, and for the control assays, the substrate was not added to the reaction mixes. Changes in absorbance per second were used to calculate the activities of the enzymes, as nkat/g fresh weight (FW). These enzymatic activity assays were performed according to [107]. For measurement of ascorbate peroxidase activity, the samples were incubated with 0.025 mM ascorbate and 0.5 mM H_2_O_2_ in 50 mM K_2_HPO_4_/KH_2_PO_4_ buffer, pH 7.6. The H_2_O_2_ was omitted for the control reactions. The absorbance of H_2_O_2_ was measured at 290 nm. Glutathione S-transferase activity was measured in samples incubated with 1 mM 2,4-dinitrochlorobenzene and 1 mM reduced glutathione in 100 mM K_2_HPO_4_/KH_2_PO_4_ buffer, pH 7.4. 2,4-Dinitrochlorobenzene was omitted for the control reactions. Absorbance was measured at 334 nm, with the formation of (2,4-dinitrophenyl) glutathione. The enzymatic activities were further corrected by subtracting the non-enzymatic formation of (2,4-dinitrophenyl) glutathione by including in the 96-well plate a column without any extract added.

## Figures and Tables

**Table 1:** The 10 genes with the most important contributions to separation of the grapevine samples according to the early and late growing seasons (Early/Late), and the same for their sanitary status (Uninfected/Infected), for the grapevines during infection with ‘*Ca*. P. solani’. E, early; L, late; U, uninfected; I, infected.

Gene ID	Description	Log_2_ FC			
		E-I : E-U	L-I : L-U	L-U : E-U	L-I : E-I
**Early/Late**					
*Vitvi02g01406*	Thaumatin family	6.62	3.27	5.52	2.18
*Vitvi06g01696*	Metallothionein	1.86	2.88	1.14	2.16
*Vitvi18g00740*	Granulin repeat cysteine protease family protein	0.93	1.19	1.72	1.98
*Vitvi19g00434*	Ubiquitin family protein	0.93	0.31	2.10	1.48
*Vitvi05g01756*	Pathogenesis-related protein 10	1.50	−0.27	1.91	0.13
*Vitvi02g00605*	Chloroplast β-amylase	0.56	−0.40	5.71	4.75
*Vitvi06g01697*	Metallothionein	0.55	1.02	−0.43	0.05
*Vitvi02g01341*	Cellulose synthase-like G3	0.73	1.13	4.34	4.75
*Vitvi03g00327*	Cold circadian rhythm and RNA binding 2	0.01	0.28	1.81	2.07
*Vitvi07g01690*	Cysteine proteinase1	1.04	0.96	2.05	1.98
**Uninfected/Infected**					
*Vitvi19g01871*	Metallothionein 3	−1.68	−0.47	0.26	1.48
*Vitvi02g00605*	Chloroplast β-amylase	0.56	−0.40	5.71	4.75
*Vitvi08g01245*	Rubisco activase	−1.14	−1.80	2.25	1.59
*Vitvi01g00714*	Galactinol synthase 4	−0.55	0.07	0.87	1.49
*Vitvi19g00549*	GDP-L-galactose phosphorylase vitamin C defective 5	−1.13	−1.51	0.54	0.16
*Vitvi17g00038*	CLPC homolog 1	−0.24	−1.05	2.01	1.19
*Vitvi05g00563*	Early light-induced protein 1, chloroplastic-related	0.24	−0.73	2.00	1.03
*Vitvi17g00320*	Ribulose bisphosphate carboxylase (small chain) family protein	−1.14	−1.67	−0.28	−0.81
*Vitvi19g00434*	Ubiquitin family protein	0.93	0.31	2.10	1.48
*Vitvi06g00513*	Rubisco activase	−1.48	−1.84	−0.35	−0.71

**Table 2:** The 10 miRNAs with the most important contributions to separation of the grapevine samples according to the early and late growing seasons (Early/Late), and the same for their health status (Uninfected/Infected), for the grapevines during infection with ‘*Ca*. P. solani’. E, early; L, late; U, uninfected; I, infected.

miRNA ID	Log_2_ FC			
	E-I : E-U	L-I : L-U	L-U : E-U	L-I : E-I
**Early/Late**				
*vvi-miR166c-h*	−1.24	−1.18	0.37	0.43
*vvi-miR162*	−1.05	−0.77	−0.05	0.24
*vvi-miR3623.5*	0.05	2.33	0.65	2.92
*vvi-miR3624-3p*	0.94	0.83	1.08	0.96
*vvi-miR3623.4*	−0.20	−0.67	0.83	0.35
*vvi-miR159c*	−0.34	−0.06	−0.26	0.02
*vvi-miR162.3*	−0.84	−0.50	−0.01	0.33
*vvi-miR3623-5p*	0.50	2.47	0.50	2.47
*vvi-miR159c.1*	−0.36	−0.07	−0.16	0.12
*vvi-miR3634.3*	−1.09	−1.84	−0.14	−0.89
**Uninfected/Infected**				
*vvi-miR3624-3p*	0.94	0.83	1.08	0.96
*vvi-miR3623.5*	0.05	2.33	0.65	2.92
*vvi-miR3623-5p*	0.50	2.47	0.50	2.47
*vvi-miR166c-h*	−1.24	−1.18	0.37	0.43
*vvi-miR156g.1*	−0.42	−0.18	1.74	1.97
*vvi-miR482*	−0.05	1.33	−0.05	1.34
*vvi-miR398b,c*	−0.54	−0.43	1.68	1.79
*vvi-miR166d.2*	−0.63	−1.67	2.08	1.04
*vvi-miR482.4*	0.98	0.31	1.00	0.32
*vvi-miR168.5*	1.05	0.58	1.01	0.55

## Data Availability

Project accession: PRJEB42777. mRNA samples accessions: ERS5673290, ERS5673291, ERS5673292, ERS5673293, ERS5673294, ERS5673295, ERS5673296, ERS5673297, ERS5673298, ERS5673299, ERS5673300, ERS5673301, ERS5673302, ERS5673303, ERS5673304, ERS5673305, ERS5673306, ERS5673307, ERS5673308, ERS5673309, ERS5673310, ERS5673311. sRNA samples accessions: ERS5672105, ERS5672104, ERS5672103, ERS5672102, ERS5672101, ERS5672100, ERS5672099, ERS5672098, ERS5672097, ERS5672096, ERS5672095, ERS5672094, ERS5672093, ERS5672092, ERS5672091, ERS5672090.

## Supplementary Materials

Supplementary materials can be found at https://www.mdpi.com/article/10.3390/ijms22073531/s1. Table S1. High-throughput mRNA-Seq of grapevine cv. Zweigelt uninfected and infected with ‘*Candidatus* Phytoplasma solani’ as vein-enriched leaf samples. BINCODE and NAME refer to [45]. For each mRNA sequence and sample, the differences in expression between the uninfected and phytoplasma-infected grapevines were calculated as log_2_ FC. Only mRNAs with a false discovery rate (FDR) adjusted *p*-value < 0.05 were considered as differentially expressed (Red, up-regulated; green, down-regulated). U, uninfected samples; I, samples infected with ‘*Ca*. P. solani’; R, recovered; E, early growing season; L, late growing season. Table S2. Known and novel grapevine miRNAs together with their expression levels in grapevine cv. Zweigelt uninfected and infected with ‘*Candidatus* Phytoplasma solani’ as vein-enriched leaf samples. Details of all of the identified known and novel miRNA sequences are given. C, conserved in plants; G, grapevine specific; N, novel unannotated. For previously described miRNAs, the identifiers (ID) of the identical miRNAs in miRBase are also given. For each miRNA sequence and sample, the differences in expression between uninfected and phytoplasma-infected grapevines were calculated as log_2_ FC. Only miRNAs with a false discovery rate (FDR) adjusted *p*-value < 0.05 were considered as differentially expressed. Red, up-regulated; green, down-regulated; U, uninfected samples; I, samples infected with ‘*Ca*. P. solani’; E, early growing season; L, late growing season; NO, no miRNA homologs in other plant species. Table S3. List of phasiRNAs identified in grapevines, together with their expression levels in grapevine cv. Zweigelt uninfected and infected with ‘*Candidatus* Phytoplasma solani’ as vein-enriched leaf samples. For each unique phasiRNA identifier (ID), the sequence and PHAS-producing locus are given. For each sample, the differences in expression between phytoplasma-uninfected and -infected grapevines were calculated as log_2_ FC. Only miRNAs with a false discovery rate (FDR) adjusted *p*-value <0.05 were considered as differentially expressed. Red, up-regulated; green, down-regulated; U, uninfected samples; I, samples infected with ‘*Ca*. P. solani’; E, early growing season; L, late growing season. Table S4. The novel miRNAs identified in grapevine cv. Zweigelt uninfected and infected with ‘*Candidatus* Phytoplasma solani’ as vein-enriched leaf samples. Table S5. Genes that are regulated by the 10 sRNAs that contributed the largest amount of variance explained by the first leading dimensions (LD1) according to the health status of the samples, and by the 10 sRNAs that contributed the largest amount of variance explained by the second leading dimensions (LD2) according to the early or late growing season for the samples in multidimensional scaling for sRNA expression in grapevine cv. Zweigelt uninfected and infected with ‘*Candidatus* Phytoplasma solani’ as vein-enriched leaf samples. Table S6. The grouping of genes in communities that: (i) form at the same times (i.e., early or late growing season) in the same group of grapevines (i.e., uninfected or infected with ‘*Ca*. P. solani’); and (ii) show significant disintegration (i.e., separation with significant dissipation index). U, uninfected grapevines; I, grapevines infected with ‘*Ca*. P. solani’; E, early growing season; L, late growing season. Table S7. Primers and probes used in this grapevine gene expression analysis.
